# The association of dietary patterns with endocannabinoids levels in overweight and obese women

**DOI:** 10.1186/s12944-020-01341-4

**Published:** 2020-07-06

**Authors:** Neda Lotfi Yagin, Samaneh Hajjarzadeh, Soghra Aliasgharzadeh, Fereshteh Aliasgari, Reza Mahdavi

**Affiliations:** 1 Student Research Committee, Nutrition Research Center, School of Nutrition and Food Sciences, Tabriz University of Medical Sciences, Tabriz, Iran; 2 Nutrition Research Center, School of Nutrition and Food Sciences, Tabriz University of Medical Sciences, Tabriz, Iran

**Keywords:** Endocannabinoids, Anadamide, 2-arachidonoylglycerol, Dietary pattern, Factor analysis, Obesity, Women

## Abstract

**Background:**

Higher levels of anandamide (AEA) and 2-arachidonoylglycerol (2-AG), the main arachidonic acid-derived endocannabinoids, are frequently reported in overweight and obese individuals. Recently, endocannabinoids have become a research interest in obesity area regarding their role in food intake. The relationship between dietary patterns and endocannabinoids is poorly understood; therefore, this study evaluated the association of the dietary patterns with AEA and 2-AG levels in overweight and obese women.

**Methods:**

In this cross sectional study, 183 overweight and obese females from Tabriz, Iran who aged between 19 and 50 years old and with mean BMI = 32.44 ± 3.79 kg/m^2^ were interviewed. The AEA and 2-AG levels were measured, and the dietary patterns were assessed using food frequency questionnaire. To extract the dietary patterns, factor analysis was applied. The association between AEA and 2-AG levels and dietary patterns was analyzed by linear regression.

**Results:**

Three major dietary patterns including “Western”, “healthy”, and “traditional” were extracted. After adjusting for age, physical activity, BMI, waist circumference, and fat mass, higher levels of AEA and 2-AG were observed in participants who were in the highest quintile of the Western pattern (*P* <  0.05). Also, in both unadjusted and adjusted models, significantly lower levels of AEA and 2-AG were detected in the women of the highest quintile of the healthy pattern (*P* <  0.01). Moreover, there was no significant association between “traditional” pattern and AEA and 2- AG levels in both unadjusted and adjusted models (*P* > 0.05).

**Conclusion:**

In regard with the lower levels of endocannabinoids in healthy dietary pattern, adherence to healthy pattern might have promising results in regulating endocannabinoids levels.

## Background

Overweight/obesity is one of the serious public health issues in developing countries such as Iran, and according to data about 22.5% of women and 10.5% of men are obese in Iran [1–3]. Scientific evidence suggests that chronic consumption of foods which contain large amounts of sugars and fats (i.e., the Western diet) is one of the main obesity drivers [4]. The worldwide impact of overweight/obesity and its complications indicate an urgent need to distinguish the important molecular mechanisms and metabolic targets implicated in energy balance [5]. In the past 15 years, the endocannabinoid system (ECS) has appeared as a lipid signaling system involved in the energy balance regulation, as it has control on every aspect of calorie regulation [6]. This system consists of endogenous ligands N-arachidonoyl-ethanolamide (anandamide), 2-arachidonoyl glycerol (2 AG), the cannabinoid 1 and 2 receptors, and enzymes responsible for the biosynthesis and degradation of ligands [7]. The endogenous ligands are lipid derivatives of a ω 6-polyunsaturated fatty acid, arachidonic acid (ARA), with multiple functions [8]. A growing body of evidence suggests that, overstimulation of ECS can lead to obesity and also to obesity- associated disorders and higher levels of ARA-derived AEA and 2-AG are frequently observed in the overweight and obese individuals [9, 10]. Since dietary intake of fatty acids is the main source of the endogenous cannabinoids biosynthesis in mammals, changes in nutritional status might affect the levels of EC [11, 12].

Nutrition transition, and specifically acquisition of a Western diet (large amounts of red meats, fast foods and snacks), is one of the factors that may help explaining the changes in the diet as well as obesity [8]. For example, in a study by Hall and colleagues, the consumption of ultra-processed foods led to greater energy intake and weight gain compared to unprocessed diets [13]. Furthermore, in Western diet (high-fat, high-sucrose)-induced obese rodents, both AEA and 2-AG levels increased, which was found to drive overeating [4]. Also, after oral exposure to dietary fats, the eCB levels elevated in the rodents’ small intestinal epithelium which in turn prompts food consumption, as CB1 receptors blockade pharmacologically in the small intestine suppressed food intake exactly before sham-feeding [14, 15]. Researchers have recently focused on dietary patterns for assessing the relationships between diet and diseases [16, 17]. It is an approach with more precise diet assessment, which provides more detailed data than analyzing one nutrient or food, as they are usually consumed with each other [18]. Although the potential roles of endocannabinoids and their respond to energy balance have been recognized highly, the important effect of different kinds of diets on endocannabinoids levels are largely unknown yet [11]. To the best of our knowledge, no study has assessed the association between overall dietary patterns and endocannabinoids levels in the overweight/ obese females. This study was conducted to examine the association of the dietary patterns with AEA and 2-AG levels in overweight and obese women.

## Material and methods

### Study participants

**Fig. 1:**
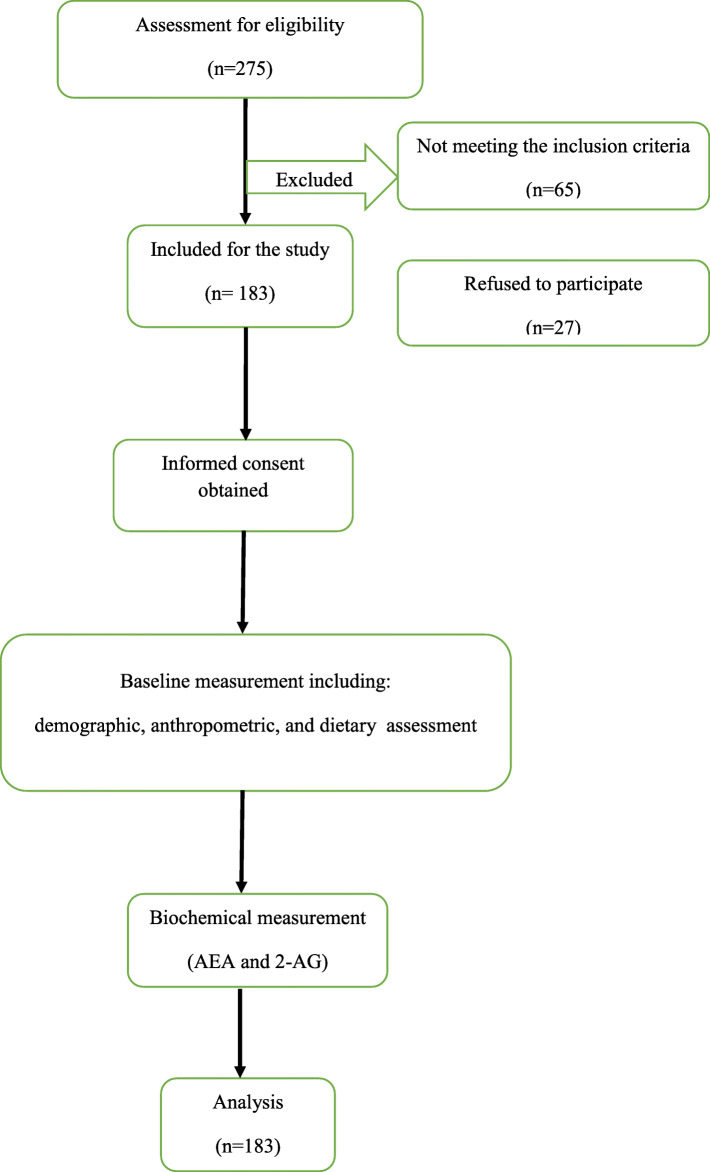
Flowchart of study design

This cross-sectional study was carried out on 183 overweight and obese women who lived in Tabriz during October 2017 to February 2018 (Fig. 1). Premenopausal women aged between 19 and 50 years old and BMI between 25 to 40 kg/m^2^ were recruited through announcements and flyer distribution in health care centers (Table 1). The subjects free of any chronic diseases such as diabetes, kidney and liver disease were included in the study. Additionally, pregnancy or lactating, consumption of any medicine affecting appetite such as antidepressant drugs as well as steroids, significant weight loss during last 3 months were also the exclusion criteria. The International Physical Activity Questionnaire (IPAQ) [19] was applied to evaluate the individuals’ physical activity and the results were presented as “low”, “moderate”, and “high”. The study protocol was approved by the Ethics Committee of Tabriz University of Medical Science (IR.TBZMED.REC.1396.620). A comprehensible consent form was signed by each participant.

**Table 1:** Participants’ demographic, anthropometric, and laboratory data

Variables	Mean ± SD
Age (year)	34.23 ± 8.22
Weight (kg)	83.12 ± 11.43
Height (cm)	159.93 ± 5.57
BMI (kg/m^2^)	32.44 ± 3.79
Waist Circumference (cm)	101.13 ± 9.04
Fat mass (kg)	34.13 ± 7.82
AEA (ng/mL)	4.54 ± 1.22
2- AG (ng/mL)	5.42 ± 1.50
Physical activity levels	n (%)
Low	101 (55.2%)
Moderate	71 (38.8%)
High	11 (6%)

All data are mean (SD) or percentage of participants; Abbreviations: *BMI* body mass index, *AEA* Anandamide, *2-AG* 2-arachidonoylglycerol.

### Anthropometric and biochemical measurements

Weight of each participant was determined in fasting state wearing light clothes and no shoes with a precision of 0.1 kg. Height was measured by a stadiometer in standing barefoot position. Body mass index was calculated as weight (kg)/(height (cm)^2^). Also, fat mass was determined by bioelectrical impedance analysis (BC-418MA, Tanita, Japan).

Whole blood samples were collected at baseline, after a 14 h fasting and the serum and plasma samples were separated from whole blood by centrifugation at 1500 g at 4 °C for 10 min and were frozen immediately at − 80 °C until assay. Plasma samples were processed no later than 10 min. Analysis of AEA and 2-AG levels was carried out using human enzyme-linked immunosorbent assay (ELISA) kits (Hangzhou Eastbiopharm Co. Ltd., Hangzhou, Zhejiang, China) [20, 21].

### Dietary assessment

Participants’ dietary intake was determined applying a valid and reliable semi-quantitative food frequency questionnaire (FFQ) containing 147 food items (with standard portion sizes) consumed frequently by Iranians [22–24]. The questionnaire was completed via direct interview by trained dietitian. The women were asked to report the frequency of consumption of every food item on a daily, weekly, monthly or yearly basis. Afterward, the stated frequency for each food object was converted into a daily intake. By utilizing household measures, the serving sizes of the consumed meals were transformed into grams. Regarding the likeness of nutrient profile, each of the 147 food items were allocated to one of the 32 specified food groups (Table 2). Standard methods were applied by trained researchers for measurements of the anthropometric indices.

**Table 2:** Food groups used for dietary pattern analysis

Food or Food groups^a^	Food items
Processed meat	Sausages
Red meat	Beef, lamb, burgers
Organ meat	Brain, liver, heart, kidney, tripe, offal
Fish	All types of fish, canned tuna
Poultry	Chicken
Egg	Eggs
Low fat dairy	Low fat milk, yoghurt, dough, curd
High fat dairy	High fat milk, full fat yoghurt, chocolate milk, cheese, ice cream, clotted cream
Fruits	Different kind of fresh fruits, dry fruits, fruit conserves
Cruciferous vegetables	All types of Cruciferous vegetables
Yellow vegetables	Cooked and raw carrots, pumpkin.
Green leafy vegetable	Lettuce, Spinach
Other vegetables	All other type of vegetables
Tomato	Tomato
Legumes	Different kind of beans, peas, lentil
Potato	Cooked potato
French fries	French fries
Whole grains	Dark Iranian bread including barbari, sangak, taftoon, barely
Refined grains	White bread including lavash, baguette, rice, pasta, vermicelli
Snacks	Biscuits, crackers, chips, cheese snacks
Nuts	Roasted and salted Walnuts, peanuts, hazelnuts, pistachio, almonds, seeds
Solid oils	Hydrogenated vegetable oil, animal oil, margarine, butter, mayonnaise
Olive	Olive oil, olive
Liquid oil	All other type of liquid oils
Vegetable pickles	All type of vegetable pickles
Pastries	All types of cookies, cakes and pastries
Sugars	Different kinds of tranditional sweets, candies, honey, jam, marmalade
Tea	Tea
Coffee	Coffee
Pizza	All types of Pizza
Natural fruit juice	All types of natural fruit juices
Soft drinks	All types of cola drinks, industrial juice

^a^32 food groups based on similarity of nutrient profile

### Statistical analysis

Data were analyzed using SPSS software (SPSS Inc., Chicago, IL, version 20). The 147 food items in the FFQ were changed into daily consumption frequencies and were then categorized into 32 food groups, with regards to their consumption frequencies and nutritional characteristics (Table 2). Factor analysis and principal component analysis were applied to detect the major eating patterns [16]. The food groups that had communalities < 0.3 were excluded. Eigenvalue > 1.5, Scree plot and natural interpretation were the criteria for retention of the factors [25]. The extracted factors (dietary patterns) were named concerning the food groups that had high positive loading, were comparable to the healthy and Western dietary patterns, and also based on the existing literatures. To compute the factor score, a weighted mean of the items related to each pattern was used. For this, every item was multiplied by its corresponding loading in factor analysis. After that, the weighted mean was divided by the sum over the loadings which is named the factor score [26]. Every participant received a factor score for each identified pattern. Participants were categorized by quintile of the dietary pattern scores.

The association between nutritional patterns and AEA, 2-AG was analyzed using linear regression models in different models (Model1: unadjusted, Model2: adjusted for BMI, WC and fat mass). The statistical significance was considered at the *P* <  0.05 level.

## Results

The participants’ demographic, anthropometric, and laboratory data is presented in Table 1. The participants’ mean age and mean BMI were 34.2 ± 8.22 years old, and 32.44 ± 3.79 kg/m^2^ respectively. The 55.2% of the individuals had low levels of activity, 38.8% were moderately active and the rest of them were highly active (6%). The AEA and 2-AG levels were 4.54 ± 1.22 ng/mL and 5.42 ± 1.50 ng/mL respectively.

By the use of factor analysis, 3 major dietary patterns were extracted which were labeled as following: the healthy dietary pattern (high in other vegetables, Cruciferous vegetables, tomato, yellow vegetables, low fat dairy, green leafy vegetable, and red meat), the Western dietary pattern (high in processed meat, organ meat, pizza, processed meat, coffee, sweets, soft drinks and French fries), and the traditional dietary pattern (tea, fish, poultry, and sugar) [27] (Table 3). Totally these three factors explained 25.47% of the whole variance. The Kaiser- Mayer- Olkin value for the items was 0.63 and the Bartlett’s Test of Sphericity was significant.

**Table 3:** Factor loading matrix for the three major dietary patterns^a^ in overweight and obese women

Food or food groups	Factor 1 (Western pattern)	Factor 2 (Healthy pattern)	Factor 3 (Traditional pattern)
**Organ meat**	0.80	-	-
**Pizza**	0.75	-	-
**Processed meat**	0.69	-	-
**Coffee**	0.65	-	-
**Pastries**	0.44	-	-
**Soft drinks**	0.41	-	-
**French fries**	0.39	-	-
**Other vegetables**	-	0.77	-
**Cruciferous vegetables**	-	0.70	-
**Tomato**	-	0.68	-
**Yellow vegetables**	-	0.56	-
**Low fat dairy**	-	0.52	-
**Green leafy vegetable**	-	0.51	-
**Red meat**	-	0.34	-
**Tea**	-	-	0.67
**Fish**	-	-	0.61
**Poultry**	-	-	0.61
**Sugar**	-	-	0.57
**Percentage of variance explained (%)**	9.85	9.30	6.33

^a^Values < 0.3 were excluded for simplicity

Linear regression analysis was applied to assess the association of AEA and 2-AG across quintile of the dietary patterns (Table 4) (Suppl. Materials 1., 2.). In the case of “Western” dietary pattern, in unadjusted model, there was no significant relationship between “Western” dietary pattern and 2-AG (*P* = 0.09). However, regarding AEA, those in the highest quintile of this pattern had significantly higher levels of AEA (*P* <  0.01) in comparison to those in the lowest quintile. Additionally, after controlling age, physical activity, BMI, waist circumference, and fat mass, the *P* value decreased and the relation between “Western” dietary pattern and 2-AG became significant (*P* <  0.05) and significantly higher levels of 2-AG were observed in those who were in the highest quintile of this pattern. Concerning the “healthy” dietary pattern, in unadjusted and adjusted models, women in the highest quintile of this pattern had significantly lower AEA and 2-AG levels (*P* <  0.01) compared to those in the lowest quintile. Also, there was no significant association between “traditional” pattern and AEA and 2- AG levels in both in unadjusted and adjusted models (*P* > 0.05).

**Table 4:** Linear regression analysis of the association between AEA and 2-AG with dietary patterns quintiles

***Models***	***Dietary pattern quintile***	***Western pattern***	***Healthy pattern***	***Traditional pattern***
***AEA***		***B (95%CI)***^**a**^	***B (95%CI)***^**a**^	***B (95%CI)***^**a**^
***Model 1***	Quintile 1	reference	reference	reference
	Quintile 2	0.35 [− 0.2–0.91]	−0.24 [− 0.76–0.28]	0.15 [− 0.42–0.73]
	Quintile 3	0.16 [− 0.39–0.72]	− 0.73 [− 1.26 – (− 0.21)]	0.15 [− 0.42–0.73]
	Quintile 4	0.45 [− 0.1–1.01]	−1.24 [− 1.79 – (− 0.74)]	− 0.06 [− 0.63–0.51]
	Quintile 5	0.93 [0.37–1.49]	−1.26 [− 1.79 – (− 0.73)]	0.08 [− 0.49–0.66]
	***P value***	**< 0.01**	**< 0.01**	**0. 94**
***Model 2***	Quintile 1	reference	reference	reference
	Quintile 2	0.37 [− 0.14–0.89]	−0.06 [− 0.55–0.42]	0.19 [− 0.34–0.73]
	Quintile 3	0.10 [− 0.42–0.64]	−0.44 [− 0.94–0.05]	0.39 [− 0.14–0.93]
	Quintile 4	0.59 [0.05–1.12]	− 1.11 [− 1.60 – (− 0.61)]	0.16 [− 0.37–0.69]
	Quintile 5	0.80 [0.27–1.34]	−1.15 [− 1.63 – (− 0.67)]	0.33 [− 0.20–0.87]
	***P value***	**< 0.01**	**< 0.01**	**0.307**
***2-AG***		***B (95%CI)***^**a**^	***B (95%CI)***^**a**^	***B (95%CI)***^**a**^
***Model 1***	Quintile 1	reference	reference	reference
	Quintile 2	0.26 [− 0.44–0.96]	− 0.56 [−1.24–0.11]	0.27 [− 0.43–0.98]
	Quintile 3	0.18 [− 0.51–0.87]	− 1.05 [− 1.73 – (− 0.36)]	− 0.21 [− 0.93–0.49]
	Quintile 4	−0.12 [− 0.81–0.57]	−1.04 [− 1.72 – (− 0.36)]	0.08 [−0.62–0.79]
	Quintile 5	0.87 [0.17–1.57]	−1.38 [− 2.06 – (− 0.69)]	−0.16 [− 0.88–0.54]
	***P value***	**0.09**	**< 0.01**	**0.51**
***Model 2***	Quintile 1	reference	reference	reference
	Quintile 2	0.13 [−0.42–0.68]	− 0.26 [− 0.79–0.26]	0.42 [− 0.13–0.98]
	Quintile 3	0.14 [−0.42–0.71]	−0.55 [− 1.09 – (− 0.11)]	0.28 [− 0.26–0.84]
	Quintile 4	0.16 [− 0.40–0.72]	−0.76 [− 1.30 – (− 0.22)]	0.42 [− 0.13–0.97]
	Quintile 5	0.61 [0.04–1.18]	−1.15 [− 1.68 – (− 0.63)]	0.39 [−0.16–0.95]
	***P value***	***< 0.05***	***< 0.01***	***0.214***

*AEA* Anandamide, *2-AG* 2-arachidonoyl glycerol.

^a^B (95%CI) of linear regression analysis

Model 1: Unadjusted.

Model 2: adjusted for age, physical activity, BMI, WC and fat mass.

Refrence group is quantlie 1 and the other groups have been compared with group 1.

## Discussion

It has been proposed that, diets that are high in fat have the potential of modulating endocannabinoids levels irrespective of their FA composition and exposure to dietary fats increases the eCB production in the rodents [14, 15, 27, 28]. It appears that complete investigation of the dietary patterns can be helpful in understanding the association between diet and AEA, and 2-AG levels.

To the best of our knowledge, this study was the first study, which evaluated the association of dietary patterns with endocannabinoids levels in the overweight/obese women.

In the current study, three major dietary patterns were identified in the overweight/obese women: “Western”, “healthy”, and traditional dietary patterns. In a study by Esmaillzadeh et al., three major dietary patterns including healthy, Western, and Iranian dietary pattern were also reported in obese women [29]. Also, in nurses with premenstrual syndrome and females with metabolic syndrome, three major dietary patterns including “Western”, “healthy”, and “traditional” patterns were extracted [30, 31].

Concerning the AEA and 2-AG levels, present results are in apparent contrast with those published previously which might be due to the differences in subjects’ demographic characteristics, intervention and sample pre-treatment [32–35].

In the present study, high adherence to Western dietary pattern resulted in significantly higher levels of AEA and 2-AG, compared to high adherence to healthy dietary pattern. The positive association between the Western pattern and endocannabinoids levels could be due to the food groups components found in this dietary pattern. In this pattern, organ meat, processed meat, pizza, French fries, and soft drinks were dominant. There is notable shift to Western dietary pattern consumption, greatly loaded in red meats, fast foods, and soft drinks in developing countries such as Iran [36]. Moreover, the prevalence of high fat diets (~ 40% of energy) is globally rising due to their palatability and also the fats low cost [37, 38].

ECS are lipid mediators and their biosynthesis can be modified directly by dietary fat intake [4, 39–41]. In animals, diets that are high in fat prompt binge eating behaviors [39] and lead to significantly elevated levels of AEA, 2-AG [40, 42], and intestinal motility [43], probably increasing stimulation of the cannabinoid receptor. Also, high fat diets caused an increase in the FA synthesis, which was partially triggered by chronic CB1^1^ activation and subsequent induction of the expression of the lipogenic transcription factor sterol regulatory element-binding protein-1c (SREBP-1c), and greater production of acetyl coenzyme-A carboxylase-1, and fatty acid synthase production [42]. As a result, the fatty acid biosynthetic pathway might be indicated as a common molecular target for the central appetitive and peripheral metabolic effects of endocannabinoids. Furthermore, a decrease in MGL^2^ and FAAH^3^ activities and an increase in NAPE-PLD^4^ action have been found to cause an elevation in AEA levels in response to high fat diets in animals [44]. However, human studies about the endocannabinoid system modulation by dietary intake are very limited. In a study by Gatta-Cherifi et al., meal containing 45% energy from carbohydrate 35% lipids, and 20% protein was tested in obese and healthy subjects. They reported an increase in fasting AEA and 2-AG levels showing the chronic overstimulation of cannabinoid receptor [45].

Additionally, a high dietary intake of linoleic acid (ω-6) can raise the arachidonic acid synthesis triggering the EC production [46]. Food processing always comprises the use of a variety of vegetable oils. The addition of vegetable oils that contain a relatively high amount of ω − 6 fatty acids contributes to an excess ratio of ω − 6 to ω − 3. Surplus intakes omega-6 vegetable oils are associated with reduction of EPA/DHA incorporation into cellular membranes, increasing the AEA and 2-AG production [46, 47]. Elevated levels of 2-AG in the whole brain and in the plasma of adults and developing animals were observed in the rats deficient in ω-3; whereas, supplementation with ω-3 seems to decrease the AEA level [48, 49]. Furthermore, Alvheim et al., showed that, in a diet with 60% of energy from lipids, rising energy from linoleic acid from 1 to 8% led to an elevation in AA in the red blood cells and liver, and also a subsequent 3-fold increase in both AEA and 2-AG [50].

At the end, it is noteworthy to point out that the higher levels of AEA and 2-AG levels in Western dietary pattern might lead to further pathological conditions as ECS dysregulation has been correlated with the development of glucose intolerance, dyslipidemia, and obesity; phenomena that are often accompanied by a myriad of neuroendocrine changes which may play a causative role in ECS dysregulation determination [51–56].

### Study strengths and limitations

Researchers have largely focused on the macronutrient portions; whereas, the association of dietary patterns with AEA and 2-AG levels was evaluated here, which can be regarded as the main strength point of the present study. However, the presented findings should also be interpreted in light of some limitations as follows: the cross-sectional design, which made it impossible to demonstrate the causality of the interactions. A FFQ with standard portion sizes was applied to estimate the food intakes, in which the measurement error (such as over -reporting or under-reporting of food intakes purposely or unintentionally) might not be precluded and might contain inaccuracies. Also, evaluation of the association of Iranian major dietary patterns with endocannabinoids levels might prevent the generalizability of data. In present study only premenopausal women were included and since menstrual cycle can affect the AEA and 2-AG levels, this issue therefore should be taken into consideration in further studies.

## Conclusions

In conclusion, three major dietary patterns were extracted in this study and the Western dietary pattern was associated with increased levels of endocannabinoids, while the healthy dietary pattern was associated with decreased AEA and 2-AG levels. Consequently, adherence to healthy dietary pattern might have promising results in regulating endocannabinoids levels. However, more longitudinal studies with dietary behaviors evaluation are required to confirm the preliminary results.

## Supplementary information

**Additional file 1: Figure S1.** The association between Western Pattern Score and AEA. **Figure S2.** The association between Healthy Pattern Score and AEA. **Figure S3.** The association between Traditional Pattern Score and AEA. **Figure S4.** The association between Western Pattern Score and 2-AG. **Figure S5.** The association between Healthy Pattern Score and 2-AG. **Figure S6.** The association between Traditional Pattern Score and 2-AG.

**Additional file 2: Figure S1.** Linear regression analysis graph of measured and whole model predicted AEA value. (Healthy Dietary Pattern). **Figure S2.** Linear regression analysis graph of measured and whole model predicted AEA value (Western Dietary Pattern). **Figure S3.** Linear regression analysis graph of measured and whole model predicted AEA value (Traditional Dietary Pattern). **Figure S4.** Linear regression analysis graph of measured and whole model predicted 2-AG value (Healthy Dietary Pattern). **Figure S5.** Linear regression analysis graph of measured and whole model predicted 2-AG value (Western Dietary Pattern). **Figure S6.** Linear regression analysis graph of measured and whole model predicted 2-AG value (Traditional Dietary Pattern).

## Data Availability

The datasets used and/or analyzed during the current study are available from the corresponding author on reasonable request.

## Abbreviations

BMI: Body mass index
ECS: Endocannabinoid system
Anandamide: N-arachidonoyl-ethanolamide
2-AG: 2-arachidonoyl glycerol
FFQ: food frequency questionnaire
MGL: Monoacylglycerol lipase
FAAH: Fatty acid amide hydrolase
NAPE-PLD: N-acyl phosphatidylethanolamine phospholipase D

## Footnotes

1 Cannabinoid receptor type 1

2 Monoacylglycerol lipase

3 Fatty acid amide hydrolase

4 N-acyl phosphatidylethanolamine phospholipase D
