# Supplementary material for: The association of dietary patterns with endocannabinoids levels in overweight and obese women
Source: Lipids Health Dis. 2020 Jul 6;19:161. doi: 10.1186/s12944-020-01341-4 (PMC7339382; doi:10.1186/s12944-020-01341-4)
Supplement: Supplementary file 1 — Additional file 1: Figure S1. The association between Western Pattern Score and AEA. Figure S2. The association between Healthy Pattern Score and AEA. Figure S3. The association between Traditional Pattern Score and AEA. Figure S4. The association between Western Pattern Score and 2-AG. Figure S5. The association between Healthy Pattern Score and 2-AG. Figure S6. The association between Traditional Pattern Score and 2-AG. [file 12944_2020_1341_MOESM1_ESM.docx]

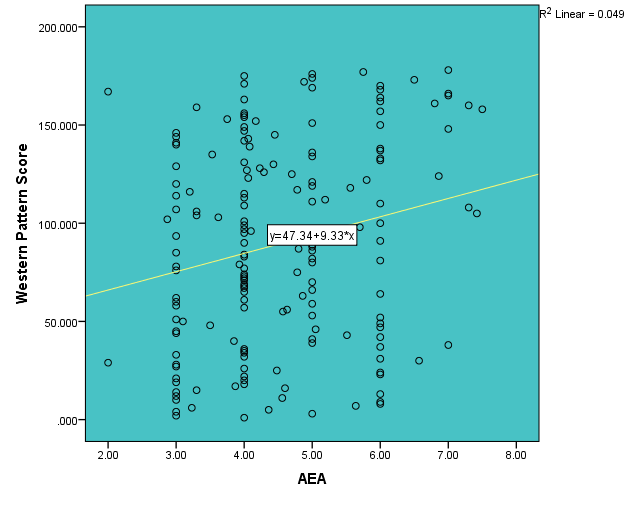


**Fig. 1 The association between Western Pattern Score and AEA**


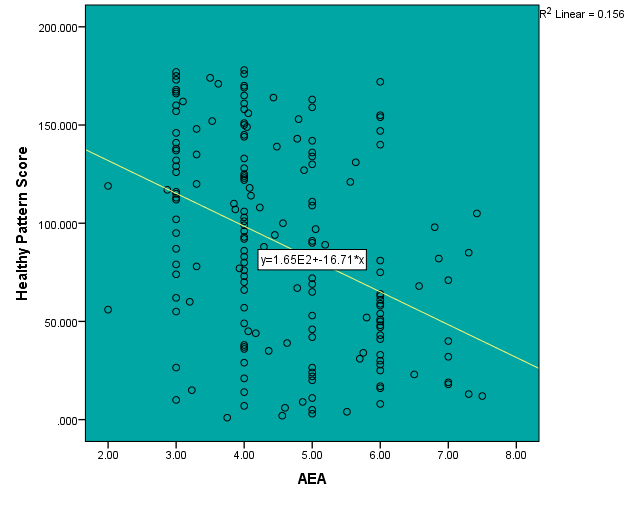


**Fig. 2 The association between Healthy Pattern Score and AEA**


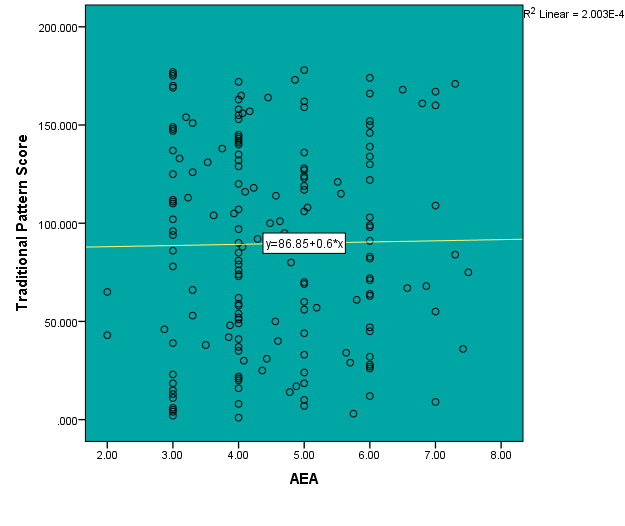


**Fig. 3 The association between Traditional Pattern Score and AEA**


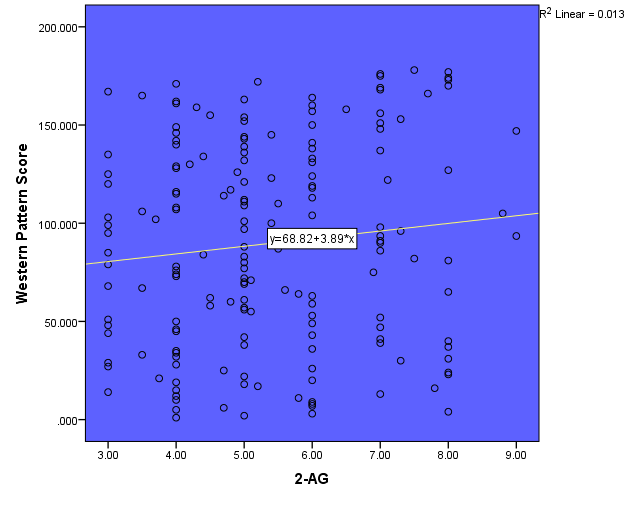


**Fig. 4 The association between Western Pattern Score and 2-AG**


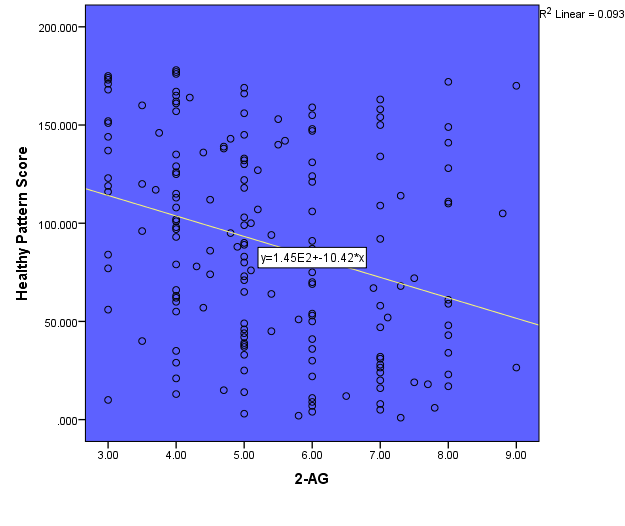


**Fig. 5 The association between Healthy Pattern Score and 2-AG**


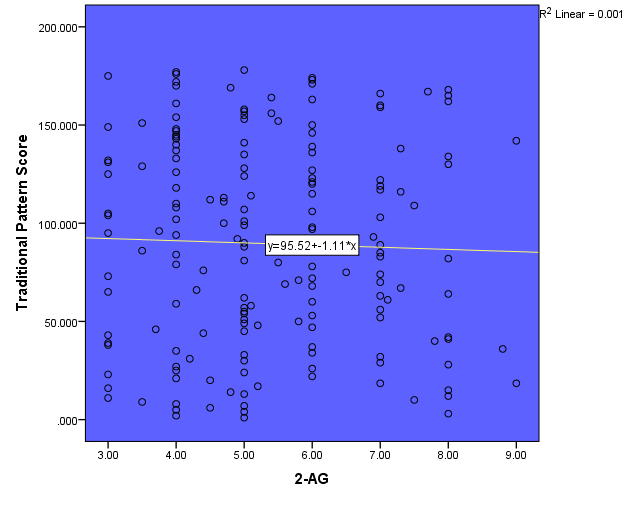


**Fig. 6 The association between Traditional Pattern Score and 2-AG**
