# Supplementary material for: The association of dietary patterns with endocannabinoids levels in overweight and obese women
Source: Lipids Health Dis. 2020 Jul 6;19:161. doi: 10.1186/s12944-020-01341-4 (PMC7339382; doi:10.1186/s12944-020-01341-4)
Supplement: Supplementary file 2 — Additional file 2: Figure S1. Linear regression analysis graph of measured and whole model predicted AEA value. (Healthy Dietary Pattern). Figure S2. Linear regression analysis graph of measured and whole model predicted AEA value (Western Dietary Pattern). Figure S3. Linear regression analysis graph of measured and whole model predicted AEA value (Traditional Dietary Pattern). Figure S4. Linear regression analysis graph of measured and whole model predicted 2-AG value (Healthy Dietary Pattern). Figure S5. Linear regression analysis graph of measured and whole model predicted 2-AG value (Western Dietary Pattern). Figure S6. Linear regression analysis graph of measured and whole model predicted 2-AG value (Traditional Dietary Pattern). [file 12944_2020_1341_MOESM2_ESM.docx]

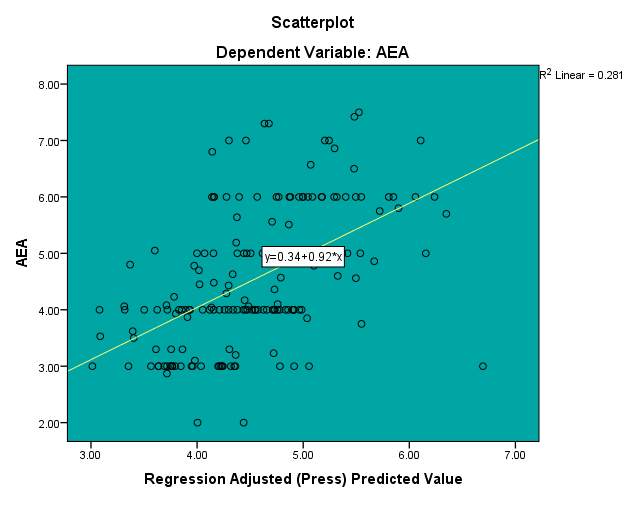


**Fig. 1 Linear regression analysis graph of measured and whole model predicted AEA value (Healthy Dietary Pattern)**

**Linear regression analysis graph of measured and whole model predicted AEA value**


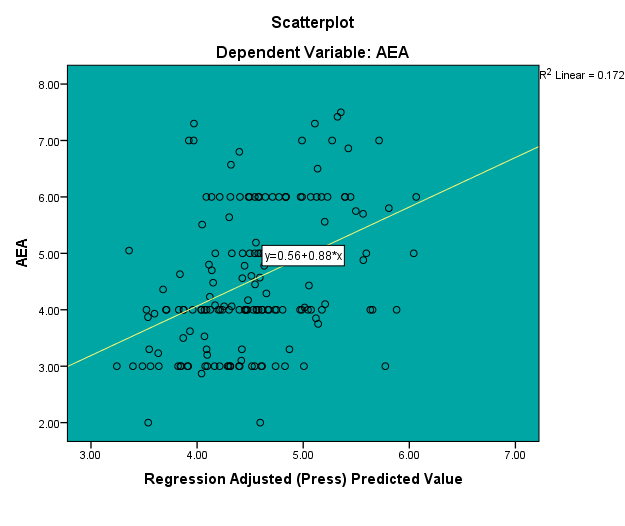


**Fig. 2 Linear regression analysis graph of measured and whole model predicted AEA value (Western Dietary Pattern)**

**Fig. 3 The association between Traditional Pattern Score and AEA**


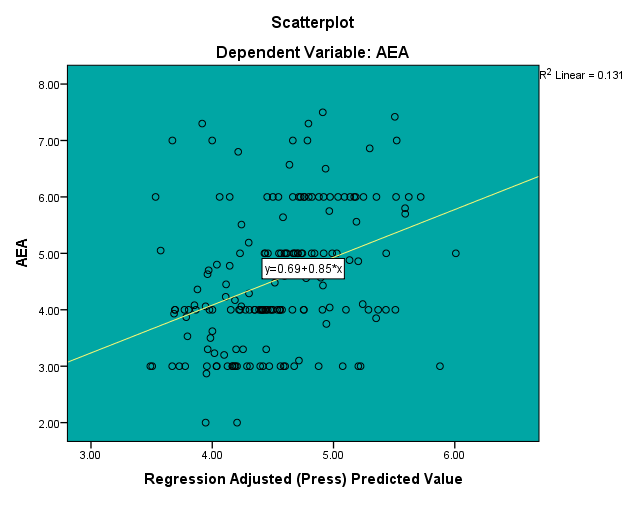


**Fig. 3 Linear regression analysis graph of measured and whole model predicted AEA value (Traditional Dietary Pattern)**


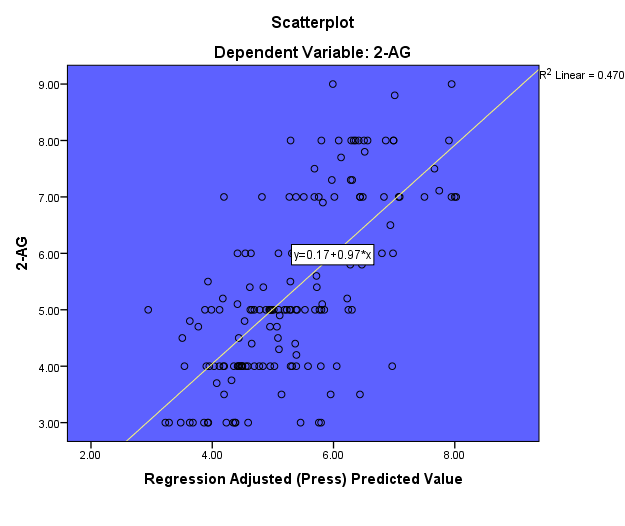

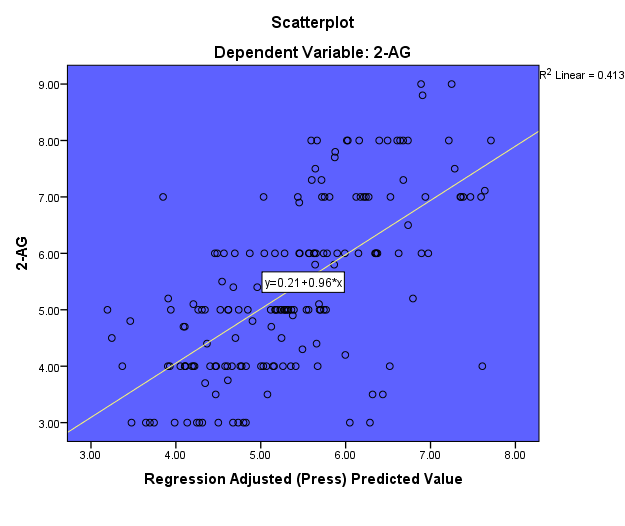

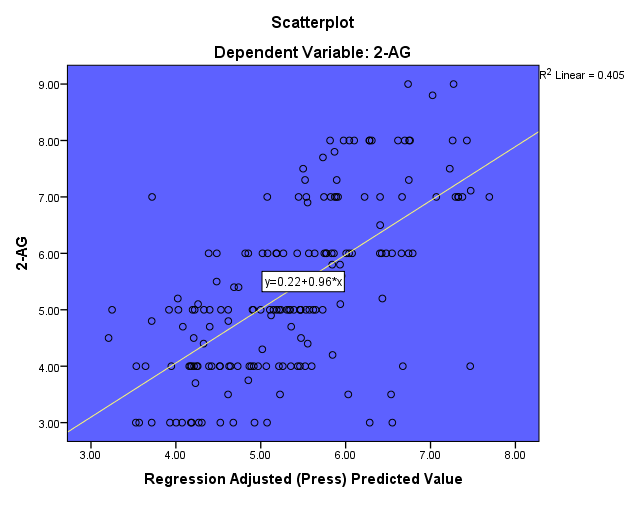


**Fig. 4 Linear regression analysis graph of measured and whole model predicted 2-AG value (Healthy Dietary Pattern)**

**Fig. 4 The association between Healthy Pattern Score and AEA**

**Fig. 5 Linear regression analysis graph of measured and whole model predicted 2-AG value (Western Dietary Pattern)**

**Fig. 6 Linear regression analysis graph of measured and whole model predicted 2-AG value (Traditional Dietary Pattern)**
